# Enamel Deproteinization or Sandblasting for Enamel Reconditioning Before Acid Etching to Enhance the Shear Bond Strength of Metallic Brackets in a Third Bonding: An In Vitro Study

**DOI:** 10.7759/cureus.66210

**Published:** 2024-08-05

**Authors:** Mohammad Saeed Al-Daher, Kinda Sultan, Mohammad Y Hajeer, Ahmad S Burhan

**Affiliations:** 1 Department of Orthodontics, Faculty of Dentistry, University of Damascus, Damascus, SYR

**Keywords:** shear bond strength, enamel deproteinization, enamel reconditioning, third bonding, first bonding, acid etching, sandblasting, enamel conditioning, bonding in orthodontics, metallic brackets

## Abstract

Background: Enamel conditioning with 37% phosphoric acid is the most common technique during orthodontic bracket bonding procedures. However, due to the repeated de-bonding of the orthodontic brackets during treatment, other methods were needed to condition the enamel surface and increase the bond strength. This study aimed to compare the effect of conditioning the enamel surface by sandblasting with aluminum oxide particles or 5.25% sodium hypochlorite gel in combination with acid etching compared to acid etching alone on shear bond strength (SBS).

Material and methods: One hundred eight extracted upper premolars were randomly divided into three groups according to the conditioning enamel surface method. After the first and second bonding of metal brackets, new metal brackets were bonded with a total-etching adhesive after enamel conditioning using different methods: acid etching only (37% phosphoric acid for 30 seconds) (AE group), sodium hypochlorite associated with acid etching (5.25% NaOCl gel for 60 seconds and then acid etching for 30 seconds) (NaOCl-AE group), and sandblasting associated with acid etching (sandblasting for five seconds and then acid etching for 30 seconds) (SB-AE group). The shear bond strengths of the brackets were tested with a universal testing machine. One-way analysis of variance (ANOVA) and Tukey's honestly significant difference (HSD) tests were used to detect significant differences in shear bond strength among groups at the third bonding. Repeated-measure ANOVA and Bonferroni's tests were used to detect significant differences in shear bond strength among the bonding attempts within each group.

Results: 5.25% sodium hypochlorite associated with the acid etching method produced significantly greater shear bond strength than sandblasting associated with acid etching and acid etching only methods at the third bonding (16.40 ± 5.80 MPa, 13.60.47 ± 6.40 MPa, and 9.90 ± 4.40 MPa, respectively; P < 0.001). However, there was no significant difference between the AE and SB-AE groups (P = 0.247). In addition, we found a significant decrease in the shear bond strength within each group after each bonding attempt.

Conclusion: Conditioning the enamel surface with 5.25% sodium hypochlorite associated with acid etching produced greater bond strength than conditioning by sandblasting associated with acid etching and acid etching only at the third bonding. The bond strength of the metal bracket decreased with increasing bonding attempts, even with the application of enamel surface conditioning methods.

## Introduction

Bracket de-bonding is a common accident during orthodontic treatment, with an incidence ranging from 1.8% to 20.1%. It may be accidental due to eating hard food, applying too much force during tooth brushing, or applying excessive orthodontic forces by the orthodontist [1]. The clinical failure rate increases with more attempts at re-bonding to the enamel surface [2]. It was found that 4% of the bracket's de-bonding occurs after the initial bonding, and this percentage increased to 14% after re-bonding for the first time and 25% after re-bonding for the second time [3]. As a result of re-bonding the brackets, the shear bond strength (SBS) of the metallic brackets decreases significantly, and this may be due to the presence of remnants of adhesive materials on the enamel surface, despite attempts to remove these materials in various ways, or to a decrease in the roughness of the enamel surface after using tungsten carbide tips [4].

Methods have been proposed, such as sandblasting, laser, and de-deproteinization, to increase the shear bond strength of the bracket to the enamel surface during re-bonding procedures to increase the roughness of the enamel surface [5]. Sandblasting with aluminum oxide particles was introduced to prepare the enamel surface before bonding procedures to increase the enamel surface's roughness and the brackets' shear bond strength. A previous study found that sandblasting provided good and clinically acceptable shear bond strength values compared to finishing burs and ultrasonic abrasive tools [6]. Similar results were found in the preparing enamel by sandblasting followed by acid etching in comparison with acid etching only during re-bonding procedures of metallic brackets, yet the shear bond strength was better than the initial bonding of the bracket [7].

Deproteinization agents, such as sodium hypochlorite, have been presented as a convenient and low-cost way to prepare the surface to remove the acquired pellicle and biofilm on the enamel, which enhances the effectiveness of the acid etching process and thus increases the stability of the brackets [8,9]. In addition, Espinosa et al. [10] reported that the area of microscopic depressions in the etched surface raised to 94.4% when the enamel was re-conditioned with 5.25% sodium hypochlorite for 60 seconds before acid etching in comparison with etching with 37% phosphoric acid alone, which led to only 45% of the full surface. The same results were found when comparing with papain gel 10% before acid etching, where the values of shear bond strength increased to 15.1 and 15.66 MPa in the sodium hypochlorite and papain groups, respectively, compared to 12.82. MPa in the acid etching group alone [11].

The previous studies that have evaluated the effect of sandblasting on re-bonding metallic orthodontic brackets were few and limited, and some of them were confined to evaluating shear bond strength only at second bonding [7]. Furthermore, none of the studies had evaluated brackets bond strength when a study tested the effect of re-conditioning the enamel surface before acid etching using sodium hypochlorite in the form of a gel on the shear bond strength at the first bond or at the second and third bond, which may have clinical benefits. It is available in the hands of orthodontists when the brackets are de-bonded [8,10,11].

Thus, this study aimed to evaluate in vitro the effects of two different enamel preparing methods: deproteinization by sodium hypochlorite gel associated with acid etching and sandblasting associated with acid etching compared to conventional preparing by 37% phosphoric acid only on metallic brackets' shear bond strength at the third bonding. The null hypothesis of this study stated that there were no differences in the shear bond strength of metallic brackets bonded with enamel prepared by sodium hypochlorite gel and sandblasting associated with acid etching compared to conventional preparation by acid etching only during the third re-bonding.

## Materials and methods

Study design and settings

This is an in vitro study design conducted between December 2023 and May 2024 at the Department of Orthodontics, Faculty of Dentistry, University of Damascus, Syria. The Local Research Ethics Committee of the Faculty of Dentistry granted ethical approval (reference number: UDDS-4242-29082022/SRC-546). The University of Damascus funded this project (reference number: 501100020595).

Sample selection and inclusion criteria

One hundred eight human upper premolars were collected. The patients whose extracted teeth were used ranged between 18 and 30 years. Patients were taken equally from males and females, with no general health condition nor previous orthodontic treatment. Inclusion criteria for teeth selection were freshly extracted premolars with intact buccal surfaces, free from white spot lesions, caries, defects, and restorations. Teeth subject to any pre-treatment chemical agents (such as fluoride varnish or hydrogen peroxide), previous orthodontic bonding, or endodontic treatments were excluded. All teeth were cleaned from periodontal debris, washed thoroughly under tap water, disinfected by immersing them in 0.1% thymol solution at room temperature for one week, and then stored in deionized water at 4°C for no longer than three months until the experiment was conducted.

Sample size estimation

Sample size estimation was done using Minitab version 19 (Minitab, Lock Haven, PA). The effect size was determined according to the observed variability of the shear bond strength in a previous study [8], employing an alpha level of 0.05 and a power of 90% and assuming the smallest difference requiring detection was 2.5 MPa. The minimal sample size was found to be 36 teeth for each group, so a total sample size of 108 teeth should be included.

Sample preparation

**Figure 1:**
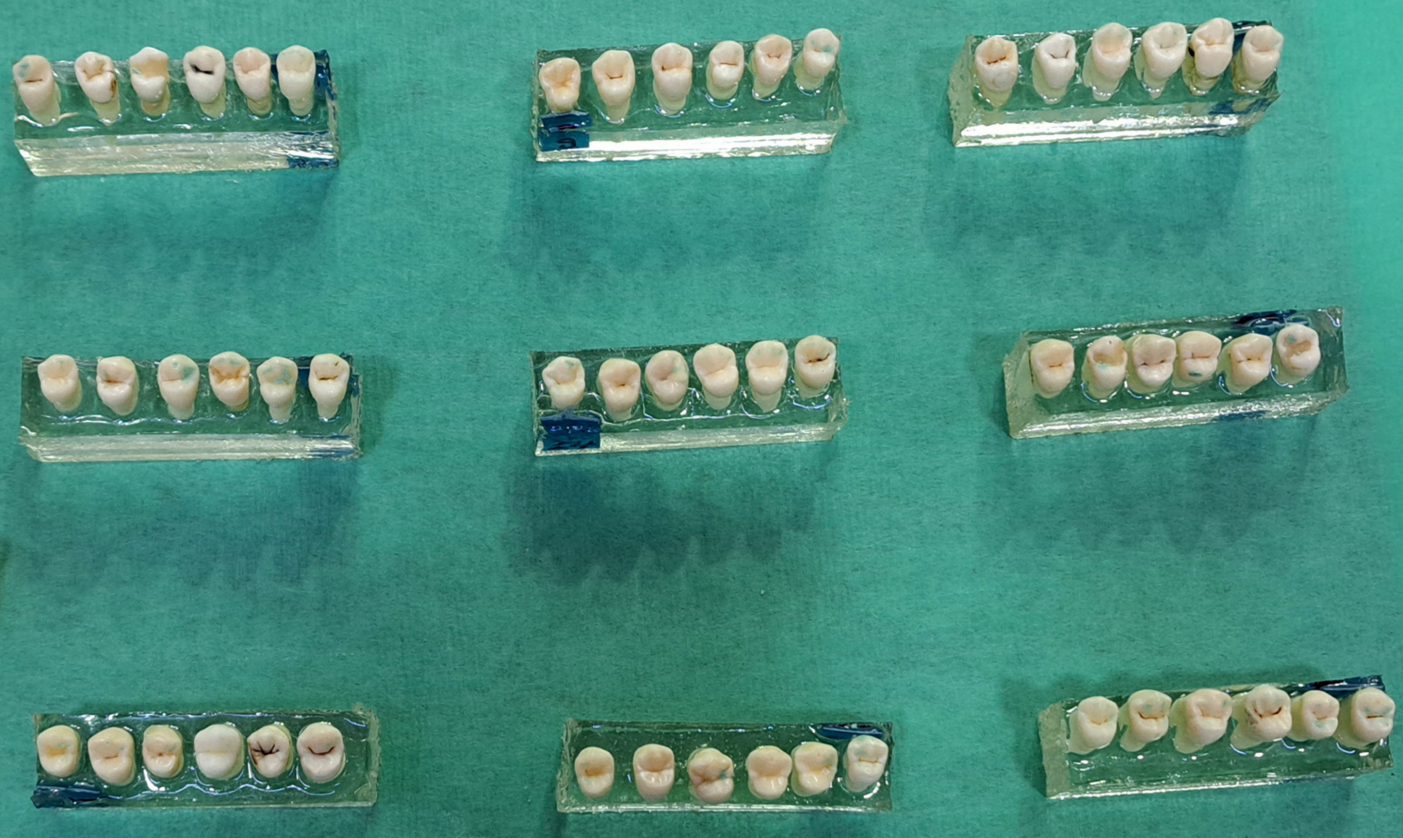
Resin molds used in the current study

Molds were made of self-curing acrylic resin for the teeth, such that each mold carried six teeth, with numbers for each tooth placed on the acrylic mold. The acrylic molds occupied the roots, which were 1-2 mm away from the cementoenamel junction so that the premolar axis was perpendicular to the lower surface of the base, which ensured that the blade of the general mechanical testing device should be parallel to the surface of the bracket base that will be bonded. The teeth were randomly divided into three groups before the third bonding (36 teeth in each group), referring to the preparing method that would be applied (5.25% sodium hypochlorite gel associated with acid etching, sandblasting associated with acid etching, and 37% phosphoric acid). In addition, all teeth within each group were given sequential numbers (from 1 to 36 written under resin blocks) to compare the shear bond strength of each specimen among re-bonding attempts. Acrylic resin molds are presented in Figure 1.

Bonding procedure and sample thermo-cycling

**Figure 2:**
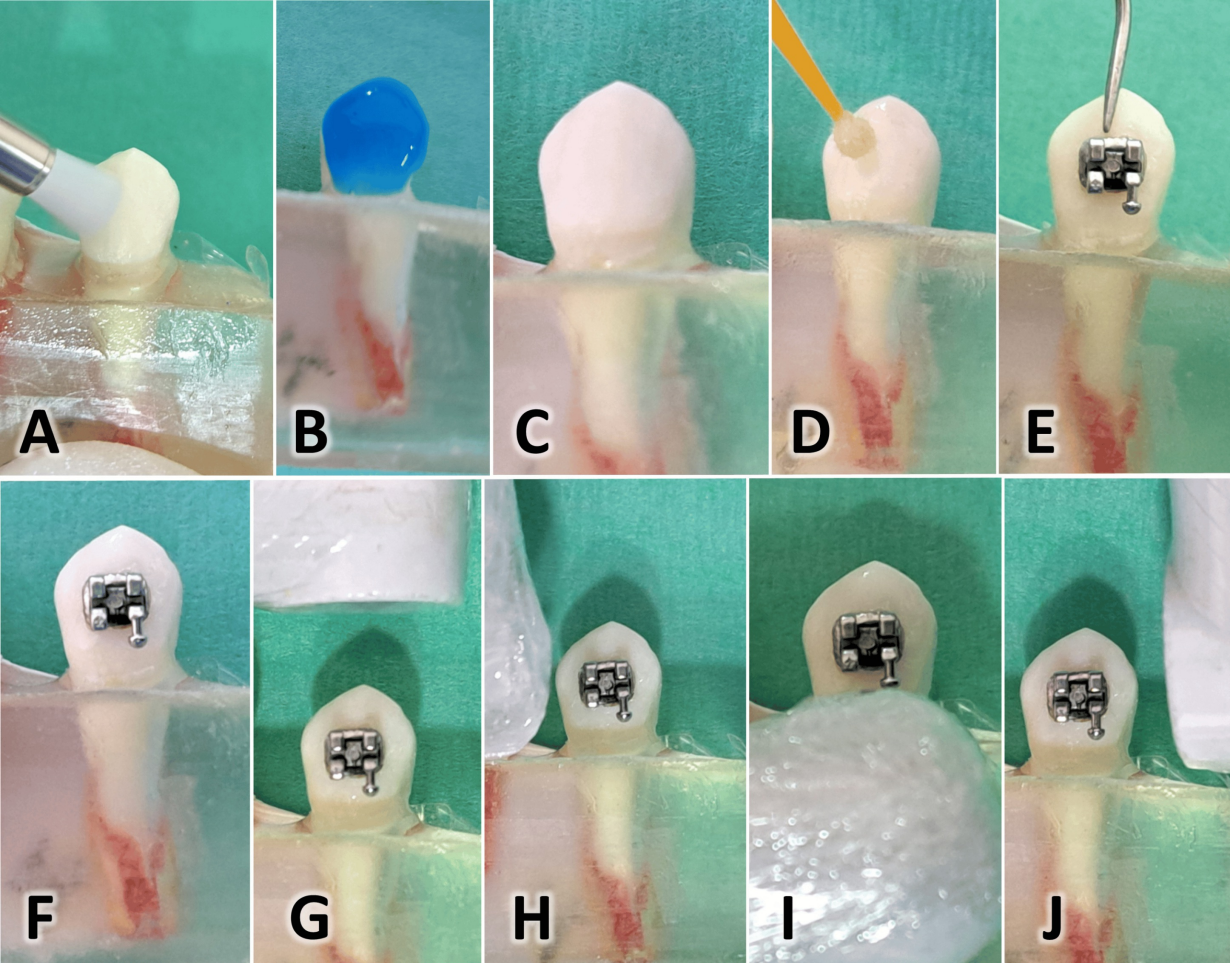
Bonding procedures A: Cleaning the buccal surface with a nylon brush bur on a low-speed handpiece. B: Etching the buccal surface with 37% phosphoric acid. C: The appearance of the buccal surface following etching. D: The application of a thin and uniform coat of bond. E: A new metal bracket was applied and pressed to its desired position using a small amount of adhesive on its base, and a dental probe removed the extra adhesive. F: Checking the position and axis of the bracket before light curing. G: The light curing tip was placed as close as possible at 45° with the buccal surface from the top. H: From the distal margin of the bracket. I: From the bottom. J: From the mesial margin of the bracket.

As performed in a previous study, the bonding procedures and thermo-cycling protocol were followed for all the samples in the first and second bonding [12]. Before etching each tooth's buccal surface, it was cleaned with water/pumice mixture, rinsed, and dried. Etching was performed for 30 seconds with 37% phosphoric acid gel (Acid Etchant Gel, American Orthodontics, Sheboygan, WI), followed by thorough rinsing and drying until a frosty white appearance of enamel was obtained. The next step was to evenly coat it with a thin layer of BracePaste® MTP Primer (American Orthodontics, Sheboygan, WI) before bonding a metal bracket (Mini Master Series® MBT Compatible 0.022, American Orthodontics, Sheboygan, WI) by using BracePasteR Adhesive (American Orthodontics, Sheboygan, WI). The metal bracket was pressed to its desired position, and a dental probe eliminated extra-adhesive material. The light guide tip of the I-Led-Plus curing light device adhesive (Woodpecker®, Wroclaw, Dolnoslaskie, Poland) was positioned at 45° as close as possible to the base margin of the bracket to cure the adhesive [12]. Bonding procedures are presented in Figure 2. After bracket bonding, teeth were incubated in distilled water at 37°C for 24 hours. Next, they were thermocycled 500 times between 5°C and 55°C with an exposure time to each bath of 20 seconds, and the transfer time between baths was five seconds [12].

After bonding, teeth underwent a 24-hour incubation period in deionized water. Next, they underwent 500 thermo-cycles between 5°C and 55°C in a Memmert BE 500 incubator (Memmert, Schwabach, Germany). This complies with the ISO standards that specify the conditions for keeping teeth when bonding tests are performed on them [13].

Shear bond strength test

Each mold was loaded into a universal testing machine (model 114, Test, Erkrath, Germany) to perform a shear force on each mold with a knife-edge blade between the bracket wings and base interface area. The blade was positioned parallel to the bracket's bond interfaces, parallel to each tooth's long axis. Shear force was applied at 1 mm/minute crosshead speed until bracket failure occurred. Values of failure loads in Newton were recorded at T1 and then converted into Mega-Pascal by dividing the failure load by the bracket base surface area, which we measured using an electronic digital caliper. The bracket's base's surface was measured and found to be 10.6 mm^2^.

Second orthodontic bracket bonding

The surface of the tooth was cleaned from the remaining adhesive material using a tungsten carbide tip (12 blades) mounted on a slow-speed retort micro-motor handpiece, washed well with a stream of water for 10 seconds, and then dried with a stream of compressed air free of moisture or oil [14].

The tooth surface was considered clean when smooth and no composite residue was visible to the naked eye under room lighting. Then, a new metal bracket was bonded at room temperature, following the previous instructions of the orthodontic adhesive system manufacturer. Then, a shear resistance test (T2) was conducted, and the value was recorded from the universal testing machine.

Third orthodontic bracket bonding

After cleaning the enamel surface, the samples were randomly distributed into three groups according to the method of preparing the enamel surface as follows: the acid etching-only group (AE group), in which enamel was prepared by 37% phosphoric acid gel only for 30 seconds; sodium hypochlorite associated with acid etching group (NaOCl-AE group), in which enamel was prepared by 5.25% sodium hypochlorite gel for one minute and then 37% phosphoric acid gel for 30 seconds; and sandblasting associated with acid etching group (SB-AE group), in which enamel was prepared by sandblasting with 50 um of aluminum oxide particles under a pressure of 60 psi for five seconds at a distance of 10 mm and an angle of 45°, followed by 37% phosphoric acid gel for 30 seconds. In the NaOCl-AE and SB-AE groups, teeth were washed well with a stream of water for 10 seconds and dried before applying 37% phosphoric acid gel. Then, a new metal bracket was bonded on the buccal surface of each tooth at room temperature following the ordinary steps of the bonding procedure.

Statistical analysis

The Statistical Package for the Social Sciences software (SPSS) version 22 (Chicago, IL) was used to perform statistical analysis. The Kolmogorov-Smirnov test was used to evaluate the normality of data distribution, and normal distributions were observed for the data concerning the shear bond strength variable. Repeated-measure analysis of variance (ANOVA) was used to detect significant differences between the three bonding attempts in each group. Bonferroni's post hoc test was used for pairwise comparisons. The three groups were compared at third bonding by one-way ANOVA test, followed by Tukey's honestly significant difference (HSD) test for post hoc comparisons.

## Results

There was a significant difference among the bonding attempts within each of the study groups (P < 0.001), and post hoc comparisons showed a significant decrease in shear bond strength among each of the bonding attempts (P < 0.001). The least decrease in the SBS was 1.1 MPa in the NaOCl-AE group compared to 1.6 MPa in the SB-AE group and 1.7 MPa in the AE group. Descriptive and analytical statistics to compare the shear bond strength among the bonding attempts within each of the study groups are presented in Table 1. Post hoc comparisons among bonding attempts in each of the study groups are presented in Table 2.

**Table 1:** Descriptive and inferential statistics of the shear bond strength among the bonding attempts within each study group †: Employing repeated-measure ANOVA *Significant difference at P < 0.001 NaOCl-AE: sodium hypochlorite plus acid etching, SB-AE: sandblasting plus acid etching, AE: acid etching only, SD: standard deviation, Min: minimum, Max: maximum, ANOVA: analysis of variance

Group	Bonding stage	Mean (MPa)	SD	Min	Max	P-value†
NaOCl-AE	First	17.50	5.80	10.6	28.2	<0.001*
	Second	17.00	6.00	10.0	28.1	
	Third	16.40	5.80	9.6	27.0	
SB-AE	First	15.20	6.10	7.0	26.9	<0.001*
	Second	14.40	6.32	5.6	25.7	
	Third	13.60	6.41	3.7	24.5	
AE	First	11.60	4.40	7.1	19.5	<0.001*
	Second	10.70	4.41	5.8	18.4	
	Third	9.90	4.40	4.1	17.7	

**Table 2:** Post hoc comparisons among bonding attempts in each study group †: Employing Bonferroni's post hoc tests *Significant difference at P < 0.001 NaOCl-AE: sodium hypochlorite plus acid etching, SB-AE: sandblasting plus acid etching, AE: acid etching only, CI: confidence interval, Min: minimum, Max: maximum

Group	Bonding stage		Mean difference (MPa)	95% CI of the difference		P-value†
				Min	Max	
NaOCl-AE	First	Second	0.50	0.20	0.70	<0.001*
	First	Third	1.10	1.00	2.10	
	Second	Third	0.60	0.40	1.00	
SB-AE	First	Second	0.80	0.40	1.20	<0.001*
	First	Third	1.60	1.00	2.60	
	Second	Third	0.80	0.40	1.20	
AE	First	Second	0.90	0.60	1.50	<0.001*
	First	Third	1.70	1.30	3.00	
	Second	Third	0.80	0.50	1.30	

Descriptive and analytical statistics to compare the shear bond strength at the third bonding among the study groups are presented in Table 3. There was a significant difference in the SBS among the three groups at the third bonding (P = 0.027). Post hoc comparisons showed that the NaOCl-AE group had a significant difference in shear bond strength than that in the SB-AE group and the AE group (P = 0.021 and P = 0.046, respectively), while there was no significant difference in shear bond strength between the SB-AE group and the AE group (P = 0.246).

**Table 3:** Descriptive and inferential statistics to compare the shear bond strength at the third bonding among the study groups †: Employing a one-way ANOVA test *: Significant difference at P < 0.05 NaOCl-AE: sodium hypochlorite associated with acid etching, SB-AE: sandblasting associated with acid etching, AE: acid etching only, NS: non-significant difference, SD: standard deviation, Min: minimum, Max: maximum, ANOVA: analysis of variance

Group	Mean (MPa)	SD	Min	Max	P-value†
NaOCl-AE	16.40	5.80	9.60	27.10	0.027
SB-AE	13.60	6.40	3.70	24.50	
AE	9.90	4.40	4.10	17.70	

## Discussion

The sample was selected from maxillary premolars only because the shear bond strength of the brackets varies significantly depending on the type of teeth tested [15]. Orthodontic adhesives within the oral cavity are exposed to temperature changes due to consuming cold and hot food and drink [16]. Different volumetric changes occur between the adhesive materials and the enamel because their linear thermal expansion is different, causing weak interface bonding. Therefore, samples from all study groups were subjected to thermal cycles to mimic the conditions of the oral cavity and were exposed to the same temperatures that they would be exposed to inside the mouth. The interval between applying the brackets and performing the tests did not exceed two weeks (during which thermal cycles were performed) [16].

Tungsten carbide spikes with 12 blades on slow-speed handpiece and with air cooling were used to remove the adhesive residue after removing the brackets after all bonding attempts. This method is considered a common and safer method for removing adhesive residues and is less likely to cause roughness to the enamel surface when compared to other methods [14]. New metal brackets were used in each bonding attempt to exclude confounding factors related to the recycled brackets, such as damage to the bracket base during bracket base cleaning procedures, and the brackets were bonded using a total-etching adhesive.

Preparation with 37% phosphoric acid is considered the most common technique during orthodontic bracket bonding procedures, which provides clinically acceptable bond strengths. Due to the repeated loss of bonding of orthodontic brackets during orthodontic treatment for several reasons, the need for other methods to prepare the enamel surface and increase the bond strength was noted, which was observed to decrease with re-bonding attempts and with less damage to the enamel surface during the re-bonding procedures. Sandblasting is considered a common method for preparing the enamel surface due to its ease and possibility of application by specialists within the dental clinic at acceptable costs, with attention to the application protocol in terms of the angle, distance of the handpiece head from the enamel surface, duration of application, air pressure applied, and size of the aluminum oxide particles used. Sandblasting was used to remove adhesive residue from the bracket base and the enamel surface when re-bonding orthodontic brackets [9,17]. On the other hand, sandblasting was used to prepare the enamel surface after removing the adhesive residue to increase the roughness of the enamel surface and increase the shear bond strength values [7,8]. It was found that sandblasting had increased the shear bond strength for re-bonding brackets [6,7]. The results of the previous studies differed regarding the angle, pressure, distance of the tip of the handle from the enamel surface, and duration of application. The safest and most effective protocol for increasing shear bond strength was chosen following these parameters: the handpiece head was tilted at a 45° angle and 10 mm away from the enamel surface, and 50-um diameter aluminum oxide particles were applied under a pressure of 60 psi for five seconds [18-20]. Sodium hypochlorite as a deproteinizing agent is a convenient, non-surgical, and low-cost method of enamel surface preparation. It effectively increased the shear bond strength of brackets at initial bonding [10,21]. With a concentration of 5.25% and an application time of 60 seconds, sodium hypochlorite was applied based on the results of previous studies [10,11,21].

This study showed that sodium hypochlorite gel associated with acid etching increased the shear bond strength of metal brackets compared to sandblasting associated with acid etching and acid etching only. In addition, we found a significant decrease in the shear bond strength after each bonding attempt within each group, and the decrease was smaller in the sodium hypochlorite associated with the acid etching group than in the sandblasting associated with the acid etching group and acid etching only group.

The results of this study agreed with previous studies that suggested preparing an enamel surface with 5.25% sodium hypochlorite before acid etching application when metallic brackets were bonded [10,11,22-24]. Also, for fluorosis enamel (which is difficult to achieve high values ​​of SBS of orthodontic bracket that bonded on it ), it has been found that the values of SBS had increased when the enamel surface had been prepared using 5.25% sodium hypochlorite [2]. Also, enamel preparation with 5.25% hypochlorite sodium before acid etching increased the values of the SBS of metallic brackets at initial bonding more than sandblasting or 37% phosphoric acid alone, which agreed with the results of this study [8]. For sandblasting, the results agreed with some of the previous studies, which have not supported enamel surface preparation with sandblasting before acid etching because they have not had a significant difference in the values of SBS [25,26]. Also, for human teeth with and without fluorosis enamel, sandblasting has not affected the values of SBS [25]. Furthermore, the results agreed with the previous studies that the mean​​ SBS of metallic brackets significantly decreased after each bonding attempt [2,22].

The results of this study differed from the results of some previous studies concerning the effect of sodium hypochlorite 5.25% on SBS when preparing the enamel surface [18,27]. Those studies have found that there was no significant difference in the SBS among the sodium hypochlorite group and the other groups, and the reason might be attributed to the study of the clinical failure rate of the brackets, which is considered less accurate compared to the shear bond strength index [27]. For sandblasting, the results of this study differed from the results of previous studies, which have found that sandblasting has increased bond strength according to the values of SBS, and the reason might be due to the difference in the type of orthodontic adhesive or the difference of parameters [7,8].

Limitations of the current study

Brackets have been bonded in the laboratory to the extracted teeth for orthodontic purposes, but the clinical failure rates of brackets have not been tested to evaluate their stability in the mouth. Upcoming studies should examine the clinical failure rate to evaluate the bonding strength.

## Conclusions

Preparing the enamel surface with 5.25% sodium hypochlorite gel associated with acid etching increases the bond strength of the metallic bracket compared to sandblasting associated with acid etching and acid etching only for re-bonding metallic brackets. This supports using this preparing method when greater bond strength is required.

Preparing the enamel surface by sandblasting with 50-um diameter aluminum oxide particles before acid etching does not affect the bond strength of the metallic bracket compared to acid etching alone for repeated bonding. The bond strengths of metal decrease with increasing bonding attempts, even with enamel surface preparation, compared to the bond strengths at the initial bonding. We recommend preparing the enamel surface by applying 5.25% sodium hypochlorite gel more than once when a bond failure occurs to obtain greater strength.
